# Trends in concussions at Ontario schools prior to and subsequent to the introduction of a concussion policy - an analysis of the Canadian hospitals injury reporting and prevention program from 2009 to 2016

**DOI:** 10.1186/s12889-018-6232-9

**Published:** 2018-11-29

**Authors:** Roman Matveev, Lauren Sergio, Jessica Fraser-Thomas, Alison K. Macpherson

**Affiliations:** School of Kinesiology & Health Science, York University, 4700 Keele Street, Toronto, ON M3J 1P3 Canada

**Keywords:** Concussion, Policy, Emergency department, Youth

## Abstract

**Background:**

Concussion is a preventable injury that can have long-term health consequences for children and youth. In Ontario, the Policy/Program Memorandum # 158 (PPM) was introduced by the Ministry of Education of Ontario in March 2014. The PPM’s main purpose is to require each school board in the province to create and implement a concussion policy. The purpose of this paper is to examine trends in school-based concussions prior to and subsequent to the introduction of the PPM.

**Methods:**

This report examined emergency department (ED) visits in 5 Ontario hospitals that are part of the Canadian Hospitals Injury Reporting and Prevention Program (CHIRPP), and compared trends over time in diagnosed concussions, and suspected concussions identified as “other head injury” in children and youth aged 4–18.

**Results:**

From 2009 to 2016 study years, there were 21,094 suspected concussions, including 8934 diagnosed concussions in youth aged 4–18. The average number of diagnosed concussions in the 5 years before the PPM was 89 concussions/month, compared to approximately 117 concussions per month after; a 30% increase in the monthly rate of concussions presenting to the ED. The total number of concussion or head injury-related ED visits remained relatively unchanged but the proportion of diagnosed concussions rose from 31% in 2009 to 53% in 2016. The proportion of diagnosed concussions in females also increased from 38% in 2013 to 46% in 2016. The percent of all diagnosed concussions occurring at schools increased throughout the study reaching almost 50% in 2016 with most injuries taking place at the playground (24%), gymnasium (22%) or sports field (20%).

**Conclusions:**

The introduction of the PPM may have contributed to a general increase in concussion awareness and an improvement in concussion identification at the school level in children and youth aged 4–18.

**Electronic supplementary material:**

The online version of this article (10.1186/s12889-018-6232-9) contains supplementary material, which is available to authorized users.

## Background

Concussions are traumatic brain injuries that can change the way the brain functions and may result in multiple transient effects including memory issues, headaches, confusion, dizziness and possible loss of consciousness [1, 2]. Children are more sensitive to the effects of concussion and are disproportionately more likely to suffer head injuries than any other age group [3]. According to recent research from the Canadian Institute for Health Information (CIHI) almost 95% of all emergency department (ED) visits for sport-related brain injuries in 2014–2015 were concussion-related [3]. There was also a 78% and a 45% increase in ED visits for 0- to 9- year olds and 10- to 17- year olds respectively [3]. Data from the US shows that sports-related concussions affect as many as 0.5–1.1 million Americans each year between the ages of 5–19, with the majority of them going to the ED [4]. Data from the provinces of Ontario and Alberta show that about 14,300 of ED visits in 2014–2015 were sports-related concussions [5]. The same data suggest that about a quarter of all brain-injuries seen in Alberta/Ontario EDs are sports-related [5]. Complications of concussions can include post-concussion syndrome (PCS), epilepsy, recurrent headaches, second impact syndrome, depression, mild cognitive impairment and chronic traumatic encephalopathy (CTE) [4, 6, 7].

Known risk factors related to pediatric concussion include being involved in a motor vehicle collision, falling, being involved in high-risk sports (e.g., football, hockey, boxing), playing without proper safety equipment or adequate supervision, and having had a concussion in the past. Studies looking at sex and age differences have been inconclusive, often showing conflicting results that vary between sports, but recent research has found increasing evidence that female athletes sustain higher rates of concussion than males in sex-comparable sports and also have a longer recovery time [8–11].

There has been a recent emphasis on concussion prevention in children and youth. In 2009, Washington State passed the Zackery Lystedt Law, named after a youth athlete who suffered a serious and debilitating brain injury in 2006 while playing football. The number of reported concussions more than doubled after the introduction of this law, possibly a result of heightened awareness and/or closer monitoring [12]. By 2013, all 50 US states had passed laws dealing with youth concussions. In Canada, on March 19, 2014, the Ministry of Education of Ontario passed the Policy/Program Memorandum # 158 (PPM) that mandates school boards to develop and implement a concussion policy [13]. The PPM consisted of a series of expectations and guidelines that each publicly funded school board in Ontario is required to adhere to when developing and implementing their own concussion policy. The primary aim of the PPM was to ensure that school boards created policies related to concussion detection and treatment, including return-to-learn guidelines by January 30, 2015 [13]. Previous attempts to enact concussion-related policy or legislation were not successful in Canada, making this PPM the first and thus far the only policy of its kind in the country. Acting in accordance with the Education Act, the PPM gives the Minister of Education the power, if necessary, to force the school boards to create and sustain the requested concussion policies [13]. An example of an school board concussion policy can be found online [14]. More recently, in June 2016, Ontario became the first province in Canada to pass a concussion policy (Rowan’s Law), which is intended to provide guidelines on the prevention, diagnosis, treatment and surveillance of head injuries and their sequelae among children and youth [15]. However, the recommendations from the Rowan’s Law committee are general recommendations that may or may not result in the enactment of legislation.

One of the issues in assessing trends in concussion is underreporting and under-diagnosis. One US based analysis shows that the rates of undiagnosed concussions range from 14.5% in a medium-sized sample of adolescent athletes, to as many as a third in a much larger sample [16, 17]. Another study found that nearly half (44.9%) of former collegiate athletes reported sustaining undiagnosed sport-related concussions [18]. Similarly, a retrospective study of concussion rates in collegiate athletes found an unreported rate of about 12% [19] while other researchers placed this number much higher, to as many as 30% [20] and even as high as 80% in certain sports such as football [21]. One problem with concussion diagnosis and reporting is the number of terms used to describe or define concussion. Frequently, these terms are used interchangeably and sometimes incorrectly, making any meaningful comparisons between studies or reports problematic [1]. Concussion reporting within Ontario schools also appears to suffer from under-reporting. While data are routinely gathered by the Ontario School Boards’ Insurance Exchange (OSBIE), in 2011 there were 634 reports of concussion or possible concussion out of 84,706 general incidence reports [22]. At about 0.7%, of the total number of incidence reports, the number of reported concussions is disproportionately lower than reported in most other studies [23, 24], thus suggesting underreporting of concussions to the insurance exchange by Ontario schools.

The past 10 years have seen an increase in ED visits for concussion in Ontario, which may be partially attributed to increased media attention, the rise of social media, numerous high profile athlete injuries and increased overall sports safety equipment, rules and regulations [25]. The objective of this study was to examine trends in school-based concussions (suspected and diagnosed) in Ontario, prior to and subsequent to the implementation of PPM 158.

## Methods

### CHIRPP overview

The Canadian Hospitals Injury Reporting and Prevention Program (CHIRPP) was selected as the data source for this study for several reasons. First, when examining data from the OSBIE, concussions appeared to be under-reported. Second CHIRPP is an injury and poisoning surveillance system that is based on data drawn from the emergency rooms of 11 pediatric hospitals and 6 general hospitals in Canada [24]. It began in 1990 and has since accumulated more than 2.8 million records nationally, with more than 80% of them being on individuals aged 19 and younger. The main objective of CHIRPP is to reduce the number and severity of injuries in Canada [26]. Any time an injured person presents to the emergency room of a participating hospital, he/she (or caregiver) is asked to complete a short one-page questionnaire that requests detailed information on the nature of their injury. These questions deal with what caused the injury, what the activity was at the time of injury, the time and location of the injury, as well as sex and age. The reverse side of the CHIRPP Form is completed by hospital staff, providing details on the nature of the injury, diagnosis, injured body part and treatment received [26]. The completed forms are entered into an electronic database by trained personnel who enter and code information on more than 40 variables and write a short overview on what transpired based on the account of the injured individual. Thus it is possible to include children who sustained an injury at school.

### Participants

Data for this study was based on five children’s hospitals in the province of Ontario. These hospitals were the *Children’s Hospital of Eastern Ontario* in Ottawa*, Hotel Dieu Hospital, including Children’s Outpatient Clinic* in Kingston*, Kingston General Hospital* in Kingston*,* The *Hospital for Sick Children* in Toronto*, and the Children’s Hospital at London Health Sciences Centre* in London, ON. Children and youth aged 4 to 18 who were treated at these hospitals between 2009 and 2016 were included in this study. The years 2009–2010 were the most recent years that included complete data in the old CHIRPP system, before it switched to an electronic version in 2011; thus the 8 years represented the most recent complete database. Individuals that were diagnosed with either a minor closed head injury or a concussion were included. Minor closed head injuries were defined as ‘suspected concussions’, signifying that even though they were not diagnosed as concussion at the time, they were still a cause for concern. A secondary analysis was also conducted on injuries that occurred in school, because the PPM only required concussion prevention and education in school boards. Beyond the switch to the electronic version in 2011, there were no known changes to the population or in the ways CHIRPP hospitals diagnosed concussions. The authors could not identify any other contextual changes that coincided with the introduction of the PPM.

### Variables of interest

This study’s primary outcome measure was a suspected or confirmed concussion, which included both minor head injuries and diagnosed concussions. The exposure variables were age, sex, month of injury, location of injury (school), and where in the school the injury took place. Age groups were separated into three categories: 4–9, 10–14 and 15–18, to align with developmental stages. The percent of diagnosed versus suspected concussions was examined for all locations and those that took place at a school, including examining where in the school the injury occurred. Information related to the school’s location was not available in CHIRPP. For the purposes of this study, we assumed that the children attended the school boards within the hospitals’ catchment areas, but we had no way to validate this assumption.

### Statistical analyses

Descriptive statistics were calculated using Statistical Analysis System (SAS) version 9.4 and Microsoft Excel. Data were only available until August 2016, making it an incomplete year. Fall 2016 was thus estimated for *graphing purposes only* using a linear approximation model. We compared the rates between diagnosed and suspected youth concussions, and for diagnosed concussions pre−/post- introduction of the PPM (March 2014), both generally and for school-incurred injuries only. Statistical significance was set at *p* < 0.05, and differences between means were based on a two-tailed t-test. In addition, a segmented linear regression analysis for an interrupted time series was performed to identify whether the introduction of the PPM #158 had an effect on the observed trends. The two independent variables were “Time” (measured in months) and “Policy” (the introduction of PPM #158). The outcome variable was the number of ED visits for diagnosed school-incurred concussions. Maximum likelihood estimation was used with an autoregressive parameters assumed given using PROC AUTOREG procedure in SAS (Additional file 1). Ethics approval was obtained from York University and the Public Health Agency of Canada.

## Results

### Diagnosed vs. suspected concussions

**Fig. 1:**
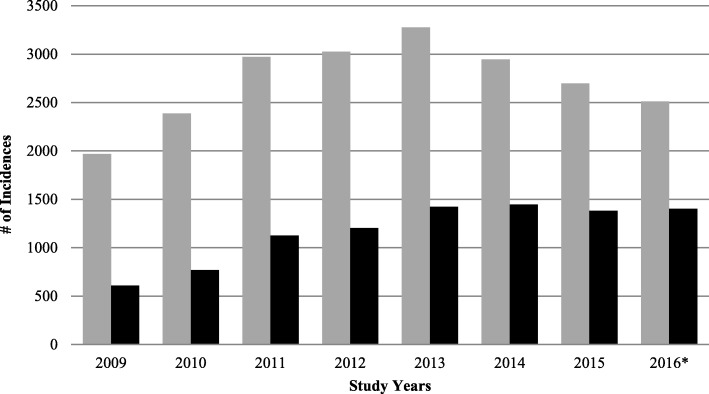
All types vs. Diagnosed Concussions. * Fall 2016 numbers were estimated using a linear approximation model

In total, between January 2009 and August 2016, there were 21,094 children and youth treated in participating EDs for head injuries, including 12,159 suspected concussion and 8935 diagnosed concussions. In total, there were 164,766 ED injury-related visits recorded in CHIRPP during this time period, with diagnosed concussions accounting for 5.4% of injury-related visits and minor closed head injuries accounting for 7.4%. In 2009, there were 610 (31%) diagnosed concussions out of 1969 suspected concussions. This number rose to 974 diagnosed out of 1822 suspected concussions (53.45%) in 2016 (Table 1). The actual number of emergency department visits for suspected concussions started to decrease in January–February 2014, just before the PPM was introduced (March 2014) but the number of diagnosed concussions remained similar (Fig. 1). The monthly average number of suspected concussions before and including the March 2014 introduction of the PPM was 232 per month. The average number of suspected concussions after the introduction of the PPM was slightly lower at 229 per month. The average rate of *diagnosed* concussions before the March 2014 PPM was about 89 concussions/month. Subsequent to the introduction of the PPM this number increased significantly by more than 30% to about 117 concussions per month (*p* < 0.001).

**Table 1:** Age and Sex Distribution of Diagnosed Concussions by Study Years

Study Year	N Diagnosed	Diagnosed as % of Suspected Concussions	Age group (years), N (%)			% Male	% In School
			4–9	10–14	15–18		
2009	610	31.0%	152 (23.1)	308 (46.9)	197 (30.0)	63.2%	30.4%
2010	770	32.3%	160 (20.0)	383 (47.8)	258 (32.2)	65.3%	26.9%
2011	1128	38.0%	227 (20.0)	553 (50.5)	335 (29.5)	64.8%	25.6%
2012	1203	39.8%	273 (22.2)	588 (47.8)	368 (30.0)	64.8%	28.2%
2013	1423	43.4%	342 (24.3)	663 (47.0)	404 (28.7)	61.8%	32.2%
2014	1445	49.1%	369 (26.0)	600 (42.1)	454 (31.9)	56.7%	35.4%
2015	1381	51.2%	325 (23.0)	638 (45.2)	449 (31.8)	56.8%	44.9%
2016*	975	53.5%	208 (24.0)	401 (46.2)	259 (29.8)	54.4%	42.3%
Total	8935	42.3%	2056 (23.0)	4134 (46.4)	2724 (30.6)	60.5%	33.8%

*Incomplete year

### Sex and age differences

The majority or 60.5% of the whole sample with diagnosed concussions were male but the difference between the sexes started to decrease in 2013–2014 (Table 1). A total of 46.4% of all diagnosed concussions were in individuals aged 10–14. The age group with the least amount of injuries, constituting 23% of the whole sample was children aged 4–9.

### Diagnosed concussions in schools

**Fig. 2:**
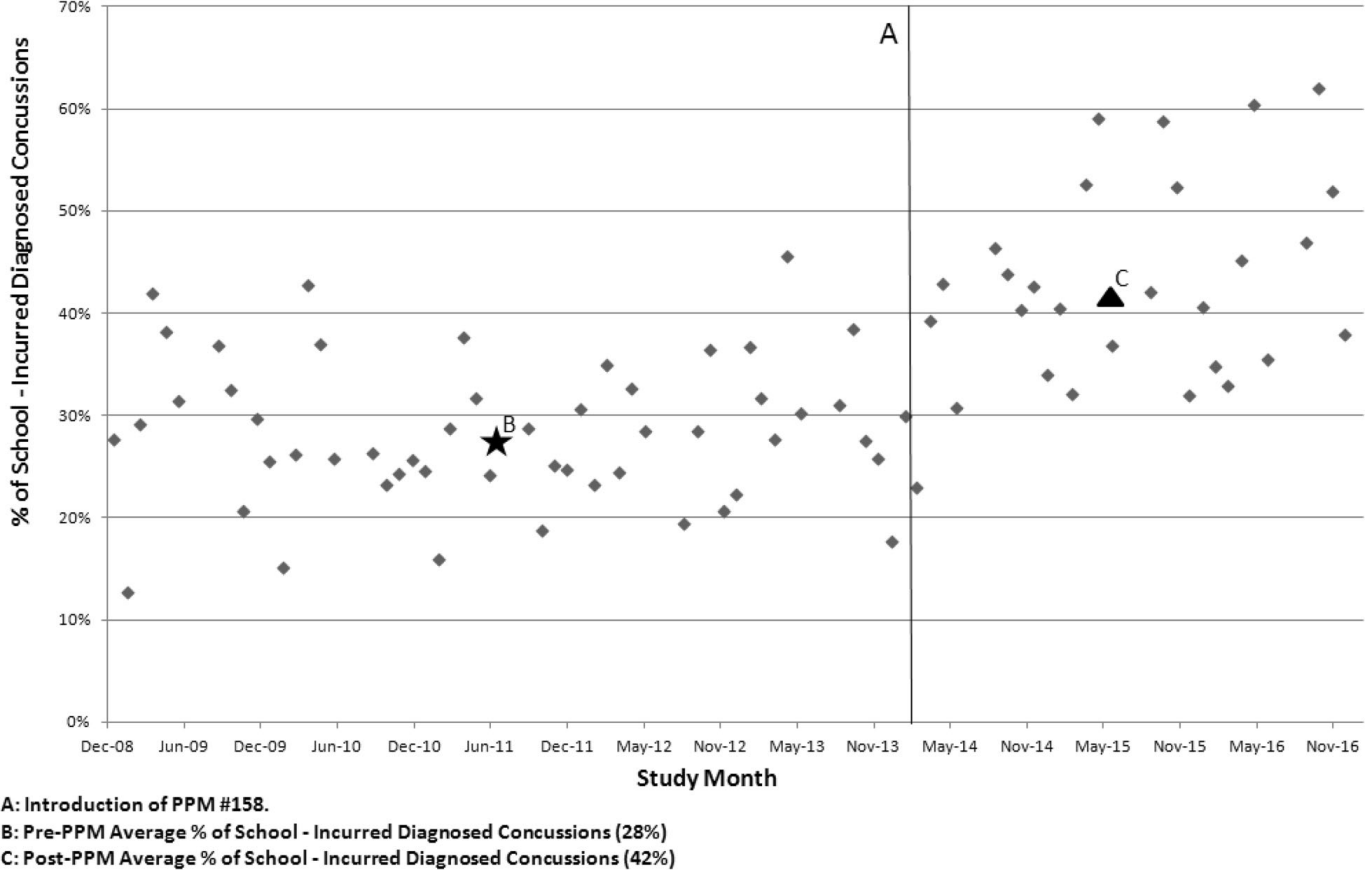
% ED Visits that were School-Incurred Diagnosed Concussions (No Summer Months)

**Fig. 3:**
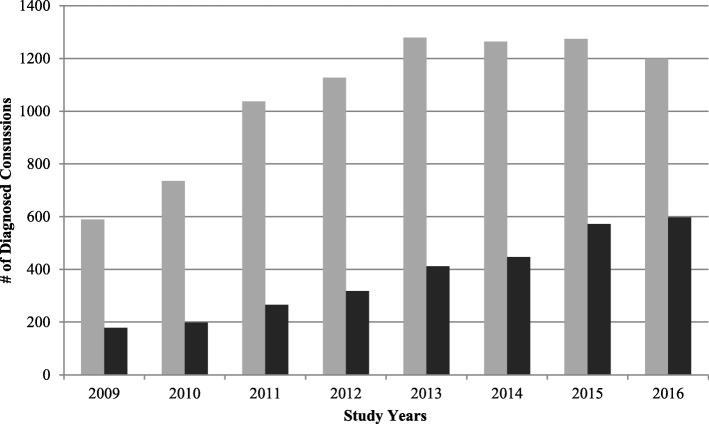
Diagnosed Concussions for All Locations vs. School

For the school-incurred diagnosed concussions data, the two summer months, July and August were removed from the analysis. The difference between *possible* (suspected) concussions before/after the PPM is not statistically significant (< 1% increase). However, the difference between diagnosed concussions was almost twofold. Prior to the PPM the number of average monthly diagnosed concussions was about 23/month, then in the months following the PPM this number rose to 44 concussions per month (excluding the estimated fall months). Figure 2 shows the percentage of ED visits for school-incurred diagnosed concussions for each month, and Fig. 3 shows the number of school-incurred diagnosed concussions relative to all locations incurred diagnosed concussions by year. Averaged out on a monthly basis, school-based concussions accounted for about 28% of all ED diagnosed concussions before and including March 2014 (PPM intro date). However, for the post-PPM months, this number rose to almost 42%. This before-after difference in the proportion of ED school-incurred diagnosed concussions was significant at *p* < 0.001. Overall school-incurred concussions accounted for more than 34% of all diagnosed concussions **(**Table 1**)**. Some months had an almost 60% school-incurred diagnosed concussion rate out of all diagnosed concussion ED visits. The proportion of all school-diagnosed concussions increased throughout the study reaching almost 50% in 2016.

### School injury location

Table 2 indicates locations within schools where diagnosed concussions occurred. The greatest proportion of all school-based, diagnosed concussions, occurred on the playground (23.7%), followed by the gymnasium (22.1%) and the sport field (20.2%). The *hallway* variable, which included the waiting area, foyer, and emergency room was combined with stairs and ramps. The lowest number of concussions happened in *other school areas*. These areas were collapsed into one category for ease of interpretation and included: the roadway, sidewalk/bus stop waiting area, school parking lot, bathroom, dining area, kitchen, office, veranda/porch, and other unspecified exterior/interior areas. Most of these areas had fewer than five diagnosed concussions over the 8-year study period.

**Table 2:** Locations in school where diagnosed concussions occurred

Location	N (%)
Playground (including swings, slides, other objects)	651 (23.7)
Gymnasium (weight room, fitness room, locker room)	609 (22.1)
Sport Field (track, sport court, rink, swimming pool)	557 (20.2)
Unknown Area (also includes missing info)	354 (12.9)
Garden/Yard (fields around the school)	310 (11.3)
Classroom (daycare indoor, activity area)	126 (4.6)
Hallway/Stairs (foyer, ramps, waiting room)	97 (3.5)
Other School Areas (bathroom, dining, office, parking lot)	48 (1.7)
Total	2752 (100.0)

### Interrupted time series analysis

**Fig. 4:**
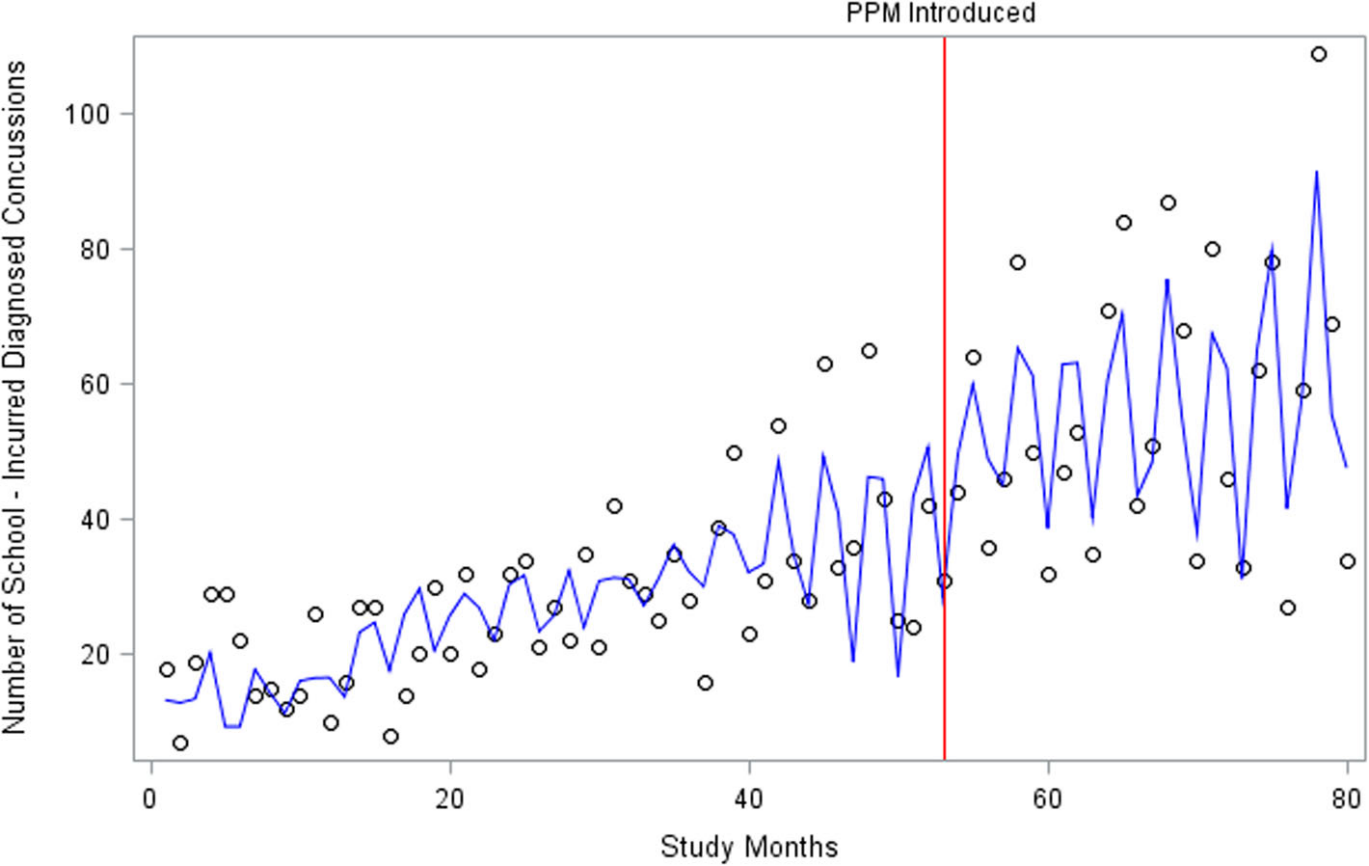
Emergency Department Visits that were School-Incurred Diagnosed Concussions

Table 3 shows the partial output from running the AUTOREG program. A maximum likelihood model was used, with backstep stepwise selection option, and using the log likelihood value for the model in the output, with assumed lag time set to 12. Overall, the rates of ED school-incurred diagnosed concussion visits were slowly trending upward at a statistically significant degree. The coefficient for the “policy” was significant, as could be also seen from Fig. 4, indicating an associated increase in the rate of ED school-incurred diagnosed concussion visits after the introduction of PPM #158.

**Table 3:** Parameter Estimates With AR Parameters Assumed Given

Variable	df	Parameter Estimate	Standard Error	t-value	Approx Pr > t
Intercept	1	12.64	1.42	8.88	< 0.0001
Policy	1	6.24	2.26	2.76	< 0.01
Time	1	0.56	0.05	11.98	< 0.0001

## Discussion

The analysis of 8 years of CHIRPP data on concussions generally, and school based concussions specifically, revealed that there was a large proportion of suspected concussions and confirmed (diagnosed) concussions compared to all-cause injury. The number of diagnosed concussions increased significantly subsequent to the introduction of PPM # 158 while the number of suspected concussions decreased.

In general, males were more likely to sustain a diagnosed concussion but after the PPM’s introduction in early 2014 the sex difference began to lessen. Recent research by Rajabali in 2011 and by the Canadian Institute for Health Information in 2016 show that males make up 50–62% of all emergency department concussion-related visits [3, 27]. The reasons for the observed decrease in the sex-based differences are unclear. Some studies suggest that there is a lack of traumatic brain injury research in women and it is unclear how undiagnosed injuries affect women [28]. Others suggest that females may sustain *more* concussions than males, but because their injuries are more often overlooked, they appear more under-diagnosed [29]. Research suggests that the recent increase in the rates of diagnosis could therefore lead to an increase in the number of confirmed concussions in women [30].

The analysis of CHIRPP data showed an increase in concussion-related ED visits until 2013 followed by a decrease by the middle of 2016 **(**Fig. 1**),** but this finding goes against recent literature on concussion trends. In a recent review of physician office and emergency department (ED) visit rates for pediatric concussion from 2003 to 2013, Zemek et al. showed a 4.4-fold (95% CI 4.37–4.45) increase per 100,000, suggesting that the rates have greatly increased, particularly since 2010 [31]. Alternatively, one study looking at various head trauma treated in U.S. emergency departments, found that concussions increased by 37.5% over the 2007–2011 study period [32] somewhat mirroring our findings of an increase in diagnosed concussions. However these reports only go to 2013 and 2011 respectively, and the data in this paper shows a similar, increasing trend up to the end of 2013 (Fig. 1). The reasons for the drop in ED visits after 2013 are unknown but could be due to changes in health service patterns (e.g., fewer patients seeking care at CHIRPP hospitals), PPM-related increases in concussion awareness or due to chance as only five hospitals had CHIRPP data, compared to other larger, injury surveillance datasets.

Since Ontario is the only province to have any concussion legislation in place it can be used as the point of departure for comparison with other provinces and territories in Canada. It is difficult to compare the rates of ED pediatric concussions in Ontario to other provinces due to variability in the settings, including potentially different diagnostic guidelines, and other factors. A recent study from the province of Quebec demonstrates an increase in rates of mild traumatic brain injuries, but the information is based on insurance records and takes into account all possible reports of medical services provided for these injuries, not just ED visits [33]. On the other hand, data from British Columbia suggests that concussion-related hospitalizations declined from 2001 to 2014 [34, 35]. A report on child and youth unintentional injury hospitalizations in Atlantic Canada (New Brunswick, Prince Edward Island, Nova Scotia & Newfoundland) between the years 2004–2013 also seems to suggest an overall decline in all-cause injury hospitalizations including head injuries and concussions [36]. Conversely, one population level study highlights the finding that the incidence of all types of injury and TBIs have increased between the years 2005 and 2014 but they do not stratify their findings by province nor do they separate concussion from other head injuries [37].

The rates of suspected versus diagnosed concussions have never been analyzed in an emergency-department setting, thus it is unclear as to why the proportion of diagnosed concussions increased with time (Fig. 1**)**. Increased awareness may have led to more confidence in diagnosing concussions rather than the more general closed head injury diagnoses.

Recent research into the effects of U.S. state concussion laws suggests that such laws are effective in improving the evaluation and detection of sports related concussions in high school students [38, 39]. Adapted from Washington State’s Zackery Lystedt Law (May 2009), these laws focus on concussion management in youth athletics via a combination of improved return-to-play and return-to-learn protocols, coach and player education, and other factors [38, 40]. One study found that the implementation of a state concussion law in Connecticut (U.S.) has led to more than a two-fold increase in the number of concussion emergency department visits, but only in high school students [38]. Similarly, not only has our study found an almost two-fold increase in the number of pre−/post- PPM school diagnosed concussions (Fig. 3**)**, but the proportion of concussions coming from a school setting has also been increasing at a constant pace (Fig. 2**)**. The majority of school-based concussions occurred either at a playground or while playing sports at the gymnasium or a sports field and this finding has been demonstrated in other Canadian studies [41].

### Limitations

CHIRPP does not represent all ED visits for youth concussions in Ontario since it only accounts for five hospitals. Thus, this study was not able to capture children that were treated elsewhere, and CHIRPP is known to have a variable capture-rate, explained elsewhere [42]. Thus, there is likely an underestimation selection bias, whereby the findings in this paper are conservative and under-representing the population at risk. However, we believe that the numbers are somewhat representative of Ontario children, especially when compared to OSBIE data. For example, in our CHIRPP school data in 2011 there were 265 confirmed, diagnosed concussions and 510 suspected concussions. OSBIE reported 634 instances of concussions or possible concussions, from all participating Ontario schools [43]. The 265 confirmed CHIRPP concussions were reported in only the five participating Ontario hospitals. That five hospitals reported more ED visits for concussion than official OSBIE reports for almost five thousand schools suggests significant underreporting or underestimation of youth concussion rates [44, 45]. Since OSBIE often works together with the Ontario Physical and Health Education Association (OPHEA) and other sports and athletics organizations across the province to develop various safety guidelines, it uses the insurance reports as the backbone or foundation for these guidelines [46]. However one report found evidence that actual youth concussion rates, at least in some sports (e.g. hockey) are *40* times greater than the officially reported OSBIE numbers [46]. It is unclear why this is the case but it raises questions about using insurance data for surveillance, and suggests that other data sources need to be considered when examining concussion trends. Only data for the five pediatric hospitals in Ontario was given to the investigators and there was no possibility to compare data from parts of Canada where there was no concussion policy or protocol in place. The CHIRPP database that was given to the primary investigators did not have a unique identifier for each hospital therefore we were unable to look at ED visits by hospital or perform a nested count analysis. Hospitals in other provinces might have had additional or different concussion regulations or criteria in place that would have made any cross-comparison results questionable. Furthermore, the diagnostic protocols in the five children’s hospitals in Ontario are supposed to be harmonized, although there is always the possibility of differing rates of concussion diagnoses. Nevertheless, Ontario hospitals use standardized diagnostic protocols that have been tested extensively by independent research organizations [31, 47].

## Conclusions

This paper examines trends in pediatric concussion in the context of Policy/Program Memorandum # 158 in the province of Ontario. PPM #158 is one important policy change that is designed to influence the reporting and diagnosis of concussions in school age children. We cautiously suggest that the PPM and subsequent school board concussion policies may have contributed to an increase in concussion awareness and improved concussion identification at the school level. This was not mirrored by a general increase in the frequency of overall head injuries, but has led to an increase in the number of diagnosed concussions. The current paper identified a few trends including an increase in diagnosed concussions, an increase in the proportion of school-based diagnosed concussions, as well as an increase in diagnosed concussion in females. PPM # 158 might have contributed to an improvement in detection of school-incurred concussions. Ongoing research, into the effectiveness of PPM 158 in terms of prevention of concussions and student, teacher, and parental awareness is required.

## Additional file

Additional file 1: Proc Autoreg Code. The code for the interrupted time series analysis in SAS (Proc Autoreg). (DOCX 10 kb)

## Abbreviations

CHIRPP: Canadian Hospitals Injury Reporting and Prevention Program
CIHI: Canadian Institute for Health Information
CTE: Chronic Traumatic Encephalopathy
ED: Emergency Department
OPHEA: Ontario Physical and Health Education Association
OSBIE: Ontario School Boards’ Insurance Exchange
PCS: Post-Concussion Syndrome
PPM: Policy/Program Memorandum
SAS: Statistical Analysis System
