# Supplementary material for: Trends in concussions at Ontario schools prior to and subsequent to the introduction of a concussion policy - an analysis of the Canadian hospitals injury reporting and prevention program from 2009 to 2016
Source: BMC Public Health. 2018 Nov 29;18:1324. doi: 10.1186/s12889-018-6232-9 (PMC6267048; doi:10.1186/s12889-018-6232-9)
Supplement: Supplementary file 1 — Proc Autoreg Code. The code for the interrupted time series analysis in SAS (Proc Autoreg). (DOCX 10 kb) [file 12889_2018_6232_MOESM1_ESM.docx]

Appendix 1

**proc** **autoreg** data=its all plots(unpack);

Model DiagnosedConcussionsSchoolOnly = policy time/

Method = ml NLAG=**12** BACKSTEP DWPROB LOGLIKL;

output out = its p=pvar r=rvar;

**run**;
